# The Health Department of Buffalo

**Published:** 1901-12

**Authors:** 


A Monthly Review of Medicine and Surgery.

*EDITOR:*

WILLIAM WARREN POTTER, M. D.

All communications, whether of a literary or business nature, books for review and exchanges should be addressed to the editor: 284 Franklin Street, Buffalo, N. Y.

Vol. XLI.—LVII. DECEMBER, 1901. No. 5.

### The Health Department of Buffalo.

WHEN the present city charter went into operation, now about ten years ago, the old board of health, with a health officer as a subordinate, was legislated out of being, and a new department under the control of a commissioner of health was created. That the system thus established has proved a benefit to Buffalo will not be disputed by any observing person. At the end of a decade, however, it may not be out of place to recall some of the important work that has been accomplished by the department. A bacteriological laboratory was established early in its career, though after considerable opposition on the part of the city legislature. At the present day, it would be next to impossible to conduct the department of health on a scientific basis without such a laboratory, presided over by a competent bacteriologist, as in the present instance.

Another innovation ordered by the present health commissioner was the reporting of contagious diseases by telephone, thus avoiding the delays entailed by other methods,—a most advantageous plan. His department, too, was the first to keep its office open at night to receive complaints of emergency and issue permits to undertakers. It may be understood, therefore, to be open continuously 365 days in the year,—nights, Sundays and holidays being included in the emergency service.

The present commissioner of health, some seven or eight years ago, succeeded in closing the Bird Island Pier inlet, which cut off a contaminated water supply to the city, thus reducing the danger of typhoid infection. With the coöperation of the State Board of Health and the State Department of Public Works, the commissioner succeeded in getting the Hamburg Canal condemned, a step that directly leads to the abatement of this perennial nuisance which is not only an eyesore to our people but a receptacle of filth and contagion.

The existing milk ordinances, which besides paying for themselves are sources of revenue, turning over to the city treasury from $1,500 to $2,000 a year, and are serving as models for other cities, were enacted at the instance of the present commissioner of health. Free public baths are also to be passed to his credit as one of the most progressive sanitary measures of this enlightened age.

The long-tube nursing bottle is another trophy hanging in the belt of this useful public officer, and through its abolition the lives of thousands of infants have been saved. Moreover, the ordinance on the subject has been adopted in other countries, notably by England. The general protective care of children in hot weather is another feature, akin to the long-tube bottle, which deserves mention.

Finally, the registration of contagious diseases to the milk routes has detected four epidemics of typhoid disease and four of scarlet fever, thus again saving hundreds of lives both of children and adults. In all this, there is economy which benefits the poor man very directly, for to prevent disease is to protect his interests, make him self-supporting, and contribute to his usefulness as a citizen. A sick person is a most expensive indulgence for a municipality to cultivate.

There are many other subjects of importance that might be dealt with in considering the health department and the good work it has done and is doing, but these are among the essential improvements which the present commissioner has put into practical operation.

A faithful, fearless, and honest public officer, who conducts his affairs efficiently upon a non-partisan principle, deserves the cordial thanks and loyal support of every intelligent citizen.

We have been led to these observations because it is known that the mayor-elect will soon be called upon to make an appointment of a commissioner of health to fill the vacancy that will occur at the end of the year, when the term of the present incumbent expires.

The health department is the only branch of the municipal government in which the medical profession has a right to advise as to its administration. It did advise as to the present heads of that department, its advice was heeded and it is not ashamed of the record that has been made—namely, a low death-rate, comparative freedom from epidemic disease, economy and non-partisanship in its conduct.

The JOURNAL has nothing but the kindest feelings and respect for the many physicians whose names have been mentioned for the succession; if one or all were connected now with the health department and its achievements had been the same we should advocate their retention. But it believes that it reflects the opinion of the great majority of the medical profession of Buffalo, as well as of many large tax-payers who are not physicians,—republicans and democrats, alike,—when it suggests the retention of the present staff of the health department intact. It believes that distinct continuity in office is necessary to the highest efficiency, that scientific intelligence in public sanitation should be the only aim, and that the action indicated herein would be both good business and good politics. We are aware that it requires great stamina of character to stand up against the political field on such a question, but it is believed that Mayor Knight is such a man.

